# Telomere erosion in NF1 tumorigenesis

**DOI:** 10.18632/oncotarget.16981

**Published:** 2017-04-09

**Authors:** Rhiannon E. Jones, Julia W. Grimstead, Ashni Sedani, Duncan Baird, Meena Upadhyaya

**Affiliations:** ^1^ Division of Cancer and Genetics, Cardiff University, Heath Park, Cardiff, CF14 4XN, UK

**Keywords:** telomere, NF1, MPNST, genetic instability, cancer

## Abstract

Neurofibromatosis type 1 (NF1; MIM# 162200) is a familial cancer syndrome that affects 1 in 3,500 individuals worldwide and is inherited in an autosomal dominant fashion. Malignant Peripheral Nerve Sheath Tumors (MPNSTs) represent a significant cause of morbidity and mortality in NF1 and currently there is no treatment or definite prognostic biomarkers for these tumors. Telomere shortening has been documented in numerous tumor types. Short dysfunctional telomeres are capable of fusion and it is considered that the ensuing genomic instability may facilitate clonal evolution and the progression to malignancy. To evaluate the potential role of telomere dysfunction in NF1-associated tumors, we undertook a comparative analysis of telomere length in samples derived from 10 cutaneous and 10 diffused plexiform neurofibromas, and 19 MPNSTs. Telomere length was determined using high-resolution Single Telomere Length Analysis (STELA). The mean Xp/Yp telomere length detected in MPNSTs, at 3.282 kb, was significantly shorter than that observed in both plexiform neurofibromas (5.793 kb; [*p* = 0.0006]) and cutaneous neurofibromas (6.141 kb; [*p* = 0.0007]). The telomere length distributions of MPNSTs were within the length-ranges in which telomere fusion is detected and that confer a poor prognosis in other tumor types. These data indicate that telomere length may play a role in driving genomic instability and clonal progression in NF1-associated MPNSTs.

## INTRODUCTION

Neurofibromatosis type 1 (NF1; MIM# 162200) is a familial cancer syndrome that affects 1 in 3,500 individuals worldwide, it is inherited in an autosomal dominant fashion and is caused by inactivation of the *NF1* tumor suppressor gene, located at 17q11.2. NF1 patients develop a variety of tumor types, including cutaneous neurofibromas, plexiform neurofibromas, malignant peripheral nerve sheath tumors, (MPNSTs), optic gliomas, phaechromocytomas, glomus tumors, gastrointestinal tumors and leukaemia [1]. While homozygous inactivation of the *NF1* gene occurs in both the benign cutaneous and plexiform neurofibromas, as well as in MPNSTs, additional genetic, biochemical and cellular changes are clearly required for malignant transformation to occur in MPNSTs. Indeed, such tumors represent a significant cause of morbidity and mortality in NF1, with patients exhibiting an 8-13% lifetime risk of developing an MPNST, often developing within a pre-existing plexiform neurofibroma, an atypical neurofibroma or a focal subcutaneous neurofibroma [2,3,4]. More than half of all MPNSTs diagnosed are found in association with NF1, with such patients usually exhibiting a poor prognosis in comparison to patients with a sporadic MPNST [5].

The presence of internal plexiform neurofibromas is strongly associated with MPNST development [6], with a high benign tumor burden also representing a risk factor for the development of MPNST. Other risk factors for the development of MPNST include the presence of an inherited gross deletion of the entire *NF1* gene region in 17q11.2 [7], previous radiation therapy [7], the presence of neurofibromatous neuropathy [8], as well as a family history of MPNSTs [9]. Unfortunately, many MPNSTs at first presentation, are already past surgical intervention as the diagnosed tumor has reached a late stage of development, with metastases commonly in the lungs, but also in the liver and brain. Thus, the 5-and 10-year survival rates for such affected NF1 individuals are only 20%–50% and 7.5%, respectively. While there are no effective treatments for MPNSTs, complete surgical excision with clear margins, if possible, is the therapy of choice, with chemotherapy and radiotherapy also being employed despite its limited effectiveness.

Several studies have analyzed aspects of tumor DNA, RNA and proteins in the search for potential molecular signatures capable of differentiating between aggressive MPNSTs and benign neurofibromas [10–21]. We have previously shown, using different DNA array platforms that MPNSTs are associated with multiple gross DNA rearrangements, with both large deletions and amplifications observed [11, 12, 15]. Recently, whole-genome/exome sequencing analyses have revealed recurrent additional genetic alterations consisting of somatic loss-of-function mutations or deletions of the Polycomb repressive complex 2 (PRC2) subunits (EED or SUZ12) in the vast majority of sporadic, NF1-associated, and radiotherapy-associated MPNSTs [19, 20, 21]. From these data it is clear that MPNST development is a complex molecular genetic process in which multiple mutations of many genes, all contributing to deregulation of multiple signaling and regulatory pathways, is to be expected.

Early detection of MPNSTs would greatly facilitate the identification and clinical management of these affected NF1 patients, however despite numerous studies no definitive information on diagnostic and prognostic biomarkers associated with MPNST development is yet available [15, 17, 18]. Telomeres are structures that cap the ends of eukaryotic chromosome protecting the natural chromosome-end from recognition by the cellular DNA damage response [22]. Telomere shortening with on-going cell division leads to the uncapping of the telomere and the initiation of a Tp53 dependent G1-S cell-cycle arrest, that provides a limit on replicative lifespan [23]. However, in the absence of a fully functional DNA damage checkpoint response, short dysfunctional telomeres can be subjected to DNA repair activities that result in the fusion of telomeres with other telomeric or non-telomeric loci [24]; the resulting di-centric chromosomes initiate cycles of anaphase-bridging, breakage and fusion that lead to large-scale genomic rearrangements of the kind observed in many tumor types [25]. Telomere length has recently emerged as a powerful prognostic and predictive marker in several tumor types, including chronic lymphocytic leukemia (CLL) [26, 27) and breast cancer [28]. Our previous studies [29] identified increased telomerase activity in high grade MPNSTs but was not detectable in diffused plexiform neurofibromas or cutaneous neurofibromas, consistent with a role for telomere dysfunction during the progression of malignancy. In this study we have focused on the possible role that telomere length may play in NF1 tumorigenesis and to evaluate the potential of high-resolution telomere length analysis as a diagnostic and prognostic tool.

## RESULTS

**Figure 1:**
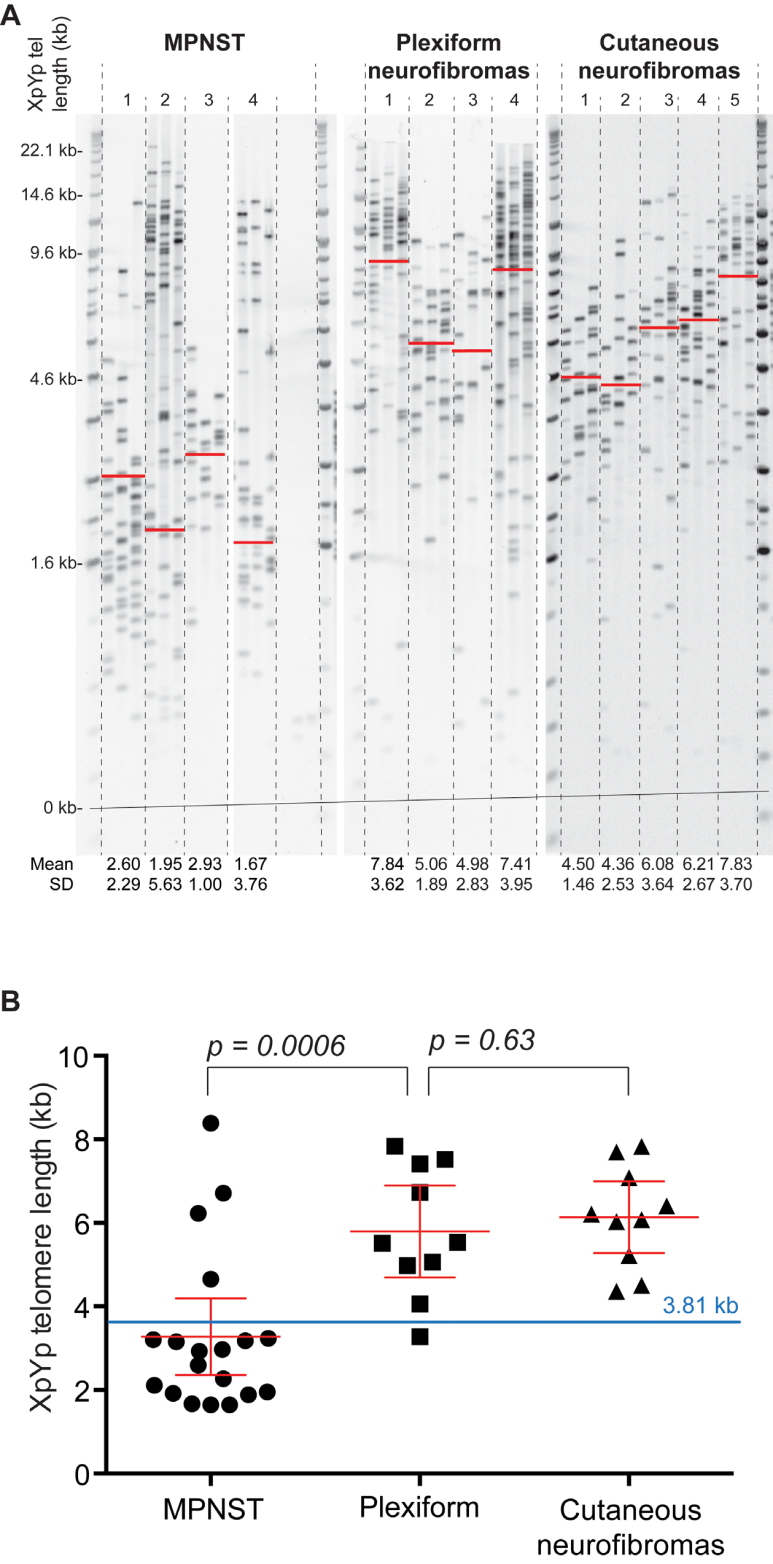
Telomere length distributions of MPNSTs are distinct from those observed in plexiform and cutaneous neurofibromas **A**., example of XpYp STELA in 4 MPNSTs, 4 plexiform neurofibromas and 5 cutaneous neurofibromas. Mean telomere length and standard deviation are detailed below the gel image and indicated with a red on the gel image. For tumors that displayed a clear bimodal distribution the mean of lower modal distribution was calculated. **B**., scatter plot depicting mean XpYp telomere length in the three different tumor types as indicated below. The 3.81 kb telomere fusion threshold, previously defined in CLL (26) is depicted in blue.

The telomere length distributions at XpYp were determined using STELA in 19 MPNSTs, 10 plexiform neurofibromas and 10 cutaneous neurofibromas (Figure 1A and supplementary figure). It was apparent from this analysis that the telomere length distributions of MPNSTs were distinct from those observed in both the plexiform and the cutaneous neurofibromas. The telomere lengths of 5 MPNSTs displayed clear bi-modal distributions; see for example, tumors 2 and 4 shown in Figure 1A. These telomere length distributions are consistent with the presence of either multiple clones within the same tumor, or the presence of normal somatic tissue within the sample. These two possibilities could not be formally distinguished and because our previous data from other tumor types [28, 30] show that tumors displaying shorter telomeres display a more aggressive phenotype, we used the mean of the lower telomere length distribution for these 5 tumors that exhibited a bimodal telomere length distribution. Overall the mean Xp/Yp telomere length detected in MPNSTs, was 3.282kb and this was significantly shorter than that observed in both plexiform neurofibromas (5.793 kb; [p == 0.0006] and cutaneous neurofibromas (6.141 kb; [p=0.0007]) (Figure 1B). The telomere length profiles observed in plexiform neurofibromas and cutaneous neurofibromas were indistinguishable [p=0.63]. The telomere erosion observed in MPNSTs was extensive, with 15 of the 19 samples (79%), displaying mean telomere length distributions that were less than the 3.81 kb telomere length threshold below which telomere fusion is detected and that confers a poor prognosis in both CLL and breast cancer [Figure 1B, 26, 28].

Using a variety of microarray platforms, we and others [10–15,5], have previously shown that DNA from MPNSTs exhibits significant large-scale genomic rearrangements, with both extensive DNA deletions and amplifications, and that these tumors also show significantly elevated levels of telomerase [29]. Owing to the limited data on samples used for telomere length profiles, we are unable to comment whether MPNSTs undergoing a period of genomic instability are driven by telomere dysfunction. A comparison between the telomere size and the genomic data derived from 13 MPNSTs from previous studies is shown in Table 1.

**Table 1:** Genomic Data on 13 NF1-associated MPNSTs

			NF1 Mutations				Target Array	
ID	Grade	Telomere Length	NF1 Germline	NF1 Somatic	p53 Mutations	Telomerase	Deleted	Amplified
1	High	Short	c2002-14C>G	LOH: I12b, IVS27, EVI20, IVS38, 3’NF, Del Array CGH	LOH	Positive	HMMR, MMP13, mTOR, NF1, OSF2, p16-INK4a, PTCH2, RB1, TP53	CCNE2, SOX10, TOP2A
2	High	Short	Exon 2,3 Del	LOH: Ex5, I12b, IVS27, EVI20, IVS38, C&CT, EW206, EW207, 3’NF1;>2.2 Mb Del Array CGH	LOH	Positive	BLBP, HMMR, HSPCA, Mki-67, MMP13, NF1, p16-INK4a, PTEN, TP53	BIRC5, CCND1, EGFR, ITGB4, TERT, TOP2A
3	Low	Short	c2497_2497delT,S8 33fsX8	Del Exon 1-4c, 6	No	Not Done	NF1, TOP2A	FOXA2
4	High	Long	Not Found	c5788_5788delC	No	Positive	NF1	CCND1, ITGB4, MMP9, PTCH2, SOX10, TERT, TP73, TSC2
5	High	Short	c3457_3460delCTC A, L1153fsX4	LOH -3’NF1: Del Array CGH	LOH	Positive	EPHA7, FN1, HMMR, Mki-67, MMP13, NF1, p16-INK4a, RB1	BIRC5, ITGB4, TERT
6	High	Short	c5234 C>G, S1745X	LOH: HHH202, I12b, IVS27, EW206	LOH	Positive	HMMR, HSPCA, LICAM2, MMP13, NF1, RASSF2	mTOR
7	High	Long	c5234 C>G, S1745X	Partial Gene Deletion - Array CGH	No	inconclusive	No Changes	No Changes
8	Low	Long	c1318C>T, R440X	LOH: HHH202, EVI20. IVS38	LOH	Negative	BLBP, EPHA7, Mki-67, MMP13, RASSF2	TOP2A
9	High	Short	Whole Gene Deletion		LOH	Not Done		
10	Low	Long?				Not Done		
11	High	Short					HGF, HMMR, HSPCA, LICAM2, MET, NF1, OSF2, p16-INK4a, RASSF2, RB1	EGFR, ITGB4, MMP9
12	Unknown	Short					MMP13, NF1, RASSF2, TP53	BIRC5, CCNE2, CCND1, EGFR, FLT4, FOXA2, HGF, HSPCA, ITGB4, MDM4, MET, mTOR, PDGFRA, S108, TERT, TOP2A, TP73
13	Unknown	Short					BLBP, HMMR, MMP13, NF1, OSF2, p16-INK4a, RB1	CDKN18/p28, FLT4, FOXM1, RASSF2

Genomic data included in this Table has been taken from previous published reports: [3,11,12,15,31,32]

LOH - Loss of heterozygosity identified either with intragenic markers or by array CGH

CGH - Comparative Genomic Hybridisation

## DISCUSSION

This study, the first to investigate the possible role of telomere length in the aetiology of NF1-associated tumors, indicates that high-resolution telomere length analysis could be important in understanding the molecular pathology of MPNSTs.

We have previously shown that the telomerase levels are elevated in MPNSTS compared to either benign cutaneous or plexiform neurofibromas [29]. To explore whether there was a potential correlation between telomere length and telomerase expression, we used the telomerase data available for MPNST 1, 2, 4, 5, 6 and 8 included in this study [29]. Interestingly, telomerase expression was observed in all tumors except for MPNST 8, which also revealed a long telomere. There is therefore no obvious relationship between telomere length and telomerase activity in MPNSTs, which is consistent with the general observations that the apparent up-regulation of telomerase activity and the presence of shorter telomeres in the majority of tumors [33, 34]. Instead we view the presence of telomerase activity and short telomeres as a marker of a tumor that has transited a telomere driven crisis, that leads to large-scale genomic rearrangements and the up-regulation of telomerase activity [24].

It is already known that shortened telomeres play an important role in tumorigenesis in many malignancies [35], including non-small cell lung cancer [36], colorectal cancer [37], prostate cancer [38], chronic lymphocytic leukemia [30], breast cancer [39], ovarian cancer [40], and several others. Our data show that the 79% of the MPNSTs analyzed displayed telomere length distributions within the length ranges at which telomere fusion is detected in CLL [26]. Stratification of CLL and Breast cancer patient cohorts based on these telomere length ranges provide powerful prognostic information [26, 28] and it is therefore possible that high-resolution telomere length analysis may also provide prognostic information in MPNSTs. Functional sequence variation in a number of genes involved in telomere maintenance have also been investigated in genetic association studies of common disease and cancer. Sequence variants located within, or immediately adjacent to the *TERT* gene, are strongly associated with several cancers, notably melanoma, breast, bladder and prostate cancers [41]. Several such *TERT* promoter mutations, that significantly increase telomerase expression, have been identified in melanomas and several other tumors. However, detailed sequence analysis of the TERT promoter in 96 MPNSTs found that <10% showed a specific C228T alteration in the *TERT* promoter [42], and these tumors were all sporadic (non-NF1-related MPNSTs). Hence, in contrast to other neuroectodermal derived malignant neoplasms, *TERT* promoter mutations occur infrequently in MPNST.

MPNSTs, like other tumors, are often extremely heterogeneous, both at the cellular and molecular levels [43, 44]. Indeed, we have shown that more than three-quarters of all MPNSTs display a degree of intra-tumoral molecular heterogeneity, as evidenced from the differences in the loss-of-heterozygosity (LOH) levels found in different regions of the same tumor [43]. The significant molecular heterogeneity evident at many different gene loci in this study clearly indicates the need to be able to integrate both molecular and morphological biomarkers in early MPNST diagnosis. The bimodal distribution of telomere length observed in different MPNSTs found in this present study, further reflects this genetic and cellular heterogeneity. The heterogeneity of telomere length profiles can reflect the clonal composition of the sample analysed, however we consider that the clear bimodal distributions observed in MPNSTs is more likely a function of purity of the sample ie the presence of both tumor and normal tissue, this cellular and molecular heterogeneity has been reported.

Many human cancers exhibit shortened telomeres and this is consistent with the increased cell division often observed during tumor progression. Telomere fusion and genomic instability, usually related to tumor progression, has been found in several tumor types [26, 37, 45]. The MPNSTs found to harbor short telomeres in this study, were already known to exhibit large-scale genomic rearrangements (Table 1) [11, 12, 15], a feature absent from other benign cutaneous and plexiform neurofibromas tumors, which predominantly show long telomeres. Such genomic instability is found in human breast cancers, where short telomeres are implicated in the progression from benign ductal carcinoma to malignant ductal carcinoma [46, 47, 28], and are also observed in several hematological cancers [48].

Genomic alterations to many other chromosomal regions have been reported in NF1-MPNSTs. Such alterations often involve the cell cycle genes *TP53*, *CDKN2A* and *RB* [1, 49], with frequent deletions of the 9p21 region containing the *CDKN2A* (p16) [50–53]. Frequent loss of the 17p13 region, encompassing the *TP53* gene, has also been found in NF1-MPNSTs [54, 55], soft tissue sarcomas and other cancers [56, 57]. Furthermore, aberrant expression of several proteins of the Rb pathway has been found in soft tissue sarcomas, including MPNSTs [53]. In the present study 5/9 MPNSTs with shortened telomeres also exhibited LOH at the *TP53* locus (Table 1). Based on our small dataset it is difficult to provide definitive correlations in NF1-associated MPNSTs between short telomeres and large-scale genomic instability. However, these observations are consistent with the hypothesis that telomere dysfunction during the progression of MPNSTs, but not plexiform neurofibromas or cutaneous neurofibromas, may drive large-scale genome instability and acquisition of specific genomic mutations that facilitate progression. Clearly further analysis is required to assess the full extent of telomere dysfunction and fusion in these tumors and to assess the prognostic and predictive potential of high-resolution telomere length analysis.

## MATERIALS AND METHODS

19 macrodissected MPNSTs were obtained from 19 unrelated patients, in addition 10 diffused plexiform and 10 cutaneous neurofibromas were also taken from 20 unrelated NF1 patients. The samples used in this study were provided by genetic centers in Cardiff (UK), London (UK), Toronto (Canada) and Hamburg (Germany). The MPNSTs were classified in accordance with the WHO classification scheme [58] and FNCLCC (French Federation Nationale des Centres de Lutte Contre le Cancer system). Reports on MPNSTs included in this study have previously been published although in order to maintain confidentiality, the identity of tumors is coded [11, 12, 15, 29, 31, 32]. DNA was extracted from fresh frozen tissue as previously reported [11] and analyzed using Single Telomere Length Analysis (STELA) [59]. This project received the full approval of the local Ethics committee. Tumor DNA was extracted and the STELA reactions at the Xp/Yp telomeres were carried out as previously described [30]. Following DNA quantification (in triplicate) by Hoechst 33258 fluorometry (BioRad, Hemel Hempstead, UK), the genomic DNA was initially diluted to 10 ng/µl in 10 mM Tris-HCl (pH 7.5) and then further diluted to 250 pg/µl in a volume of 40 µl that contained 1 µM Telorette2 linker and 1 mM Tris-HCl (pH 7.5). Multiple PCRs were carried out for each test DNA in 10 µl volumes, containing 250 pg of diluted DNA, 0.5 µM of the telomere-adjacent and Teltail primers, 75 mM Tris-HCl (pH 8.8), 20 mM (NH4)_2_SO4, 0.01% Tween-20, 1.5 mM MgCl_2_, and 0.5 U of a 10:1 mixture of Taq (ABGene, Epsom, UK) and Pwo polymerase (Roche Diagnostics, West Sussex, UK). The reactions were cycled with an MJ PTC-225 thermocycler as described previously [30]. The DNA fragments were resolved by 0.5% TAE agarose gel electrophoresis, and detected Southern hybridisation with a random-primed [α-^33^P] labelled (PerkinElmer, Coventry, UK) telomere repeat containing probe together with a probe to detect the 1 kb (Stratagene, La Jolla, CA, USA) and 2.5 kb (BioRad, Hemel Hempstead, UK) molecular weight markers. The hybridised fragments were detected by phosphorimaging with a Typhoon FLA 9500 phosphorimager (GE Healthcare, St Giles, UK). The molecular weights of the DNA fragments were calculated using the Phoretix 1D quantifier (Nonlinear Dynamics, Newcastle-upon-Tyne, UK).

Statistical comparisons were made using the nonparametric Mann Whitney tests (Prism 6).

## SUPPLEMENTARY MATERIALS FIGURES AND TABLES


