# Supplementary material for: Telomere erosion in NF1 tumorigenesis
Source: Oncotarget. 2017 Apr 9;8(25):40132–9. doi: 10.18632/oncotarget.16981 (PMC5522233; doi:10.18632/oncotarget.16981)
Supplement: Supplementary file 1 [file oncotarget-08-40132-s001.pdf]

Telomere erosion in NF1 tumorigenesis

Supplementary Material

A

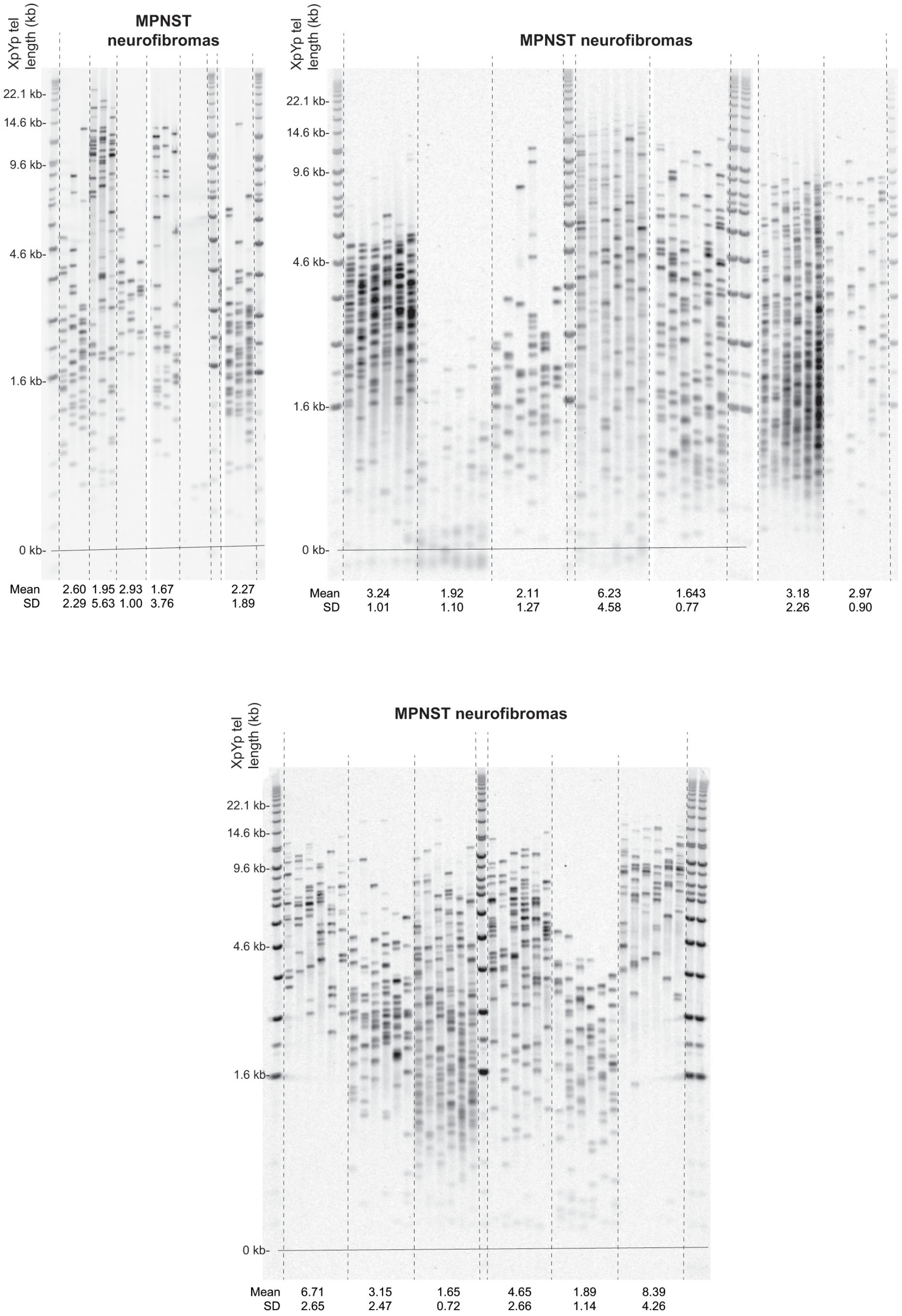

B

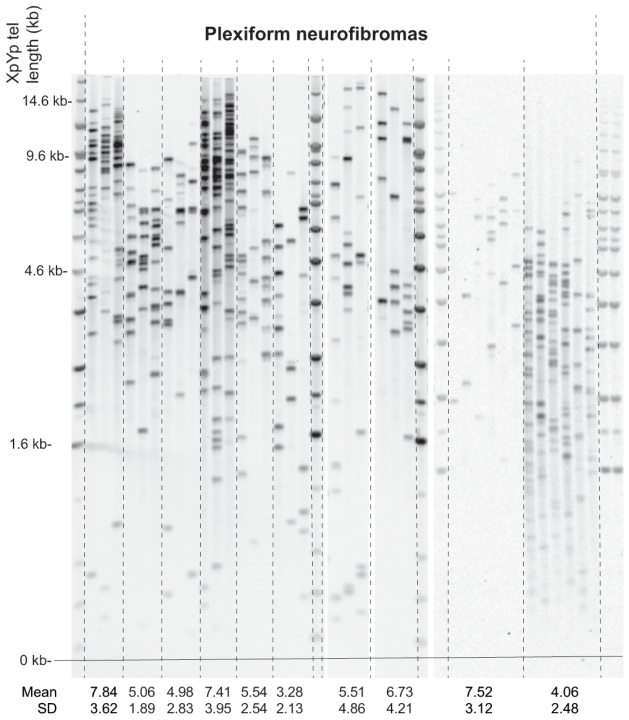

C

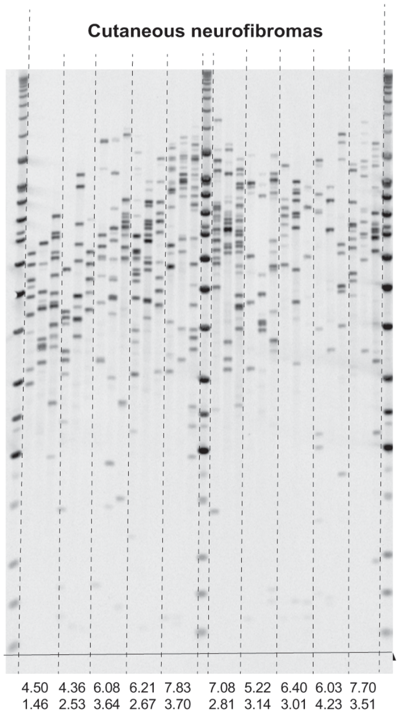

Supplementary figure.

STELA of the XpYp telomere in A, MPNST neurofibromas, B, plexiform neurofibromas, C, cutaneous neurofibromas. Mean and SD are detailed below. For tumours that displayed a clear bimodal distribution the mean of lower modal distribution was calculated.

Supplementary Table 1

ID	Grade	Location	Reference
1	High	Right lower calf	Spurlock et al, 2010
2	High	Neck	Spurlock et al, 2007
3	High	Plexiform	
4	High	Plexiform	
5	High	Plexiform	
6	High	Plexiform	
7	High	Radiation induced	
8	High	Radiation induced	
9	High	Not known	
10	Low	Right axilla	
11	High	Neck	
12	High	Left supra clavicular	
13	Not known	Not Known	
14	High	Not Known	
15	High	Not Known	
16	High	Not Known	
17	High	Not Known	
18	High	Thoracic	
19	Low	Right hamstring	
